# Medication-Overuse Headache: Update on Management

**DOI:** 10.3390/life14091146

**Published:** 2024-09-11

**Authors:** Prut Koonalintip, Katherine Phillips, Benjamin R. Wakerley

**Affiliations:** 1 Division of Neurology, Department of Internal Medicine, Prince of Songkla University, Hatyai 90110, Songkhla, Thailand; koo.prut@gmail.com; 2 Department of Neurology, University Hospitals Birmingham NHS Foundation Trust, Birmingham B15 2GW, UK; 3 Metabolic Neurology, Institute of Metabolism and Systems Research, The Medical School, College of Medical and Dental Sciences, University of Birmingham, Birmingham B15 2TT, UK; 4 Institute of Applied Health Research, University of Birmingham, Birmingham B15 2TT, UK

**Keywords:** medication-overuse headache, rebound headache, chronic migraine

## Abstract

Long-term frequent use of acute pain medication for the treatment of headaches has paradoxically been shown to increase the frequency of headaches. So-called medication-overuse headache (MOH) is particularly problematic in patients with migraine who overuse triptans and opioids. Prevention through education remains the most important management strategy. Once established, MOH can be difficult to treat. Although complete or near-complete withdrawal of acute pain medication for 8–12 weeks has been shown to benefit most patients, this can be hard to achieve. The use of OnabotulinumtoxinA and drugs that target the calcitonin gene-related peptide system for the prevention of migraines have been shown to benefit patients with MOH. Furthermore, the use of novel acute pain medication for migraines, including Gepants and Ditans, which do not cause MOH, are likely to improve patient outcomes. In this review article we examine the following: the burden of MOH; who develops MOH; the pathophysiological mechanisms; and the treatment strategies.

## 1. Introduction

In 1951, Peters and Horton identified that the daily intake of Ergotamine for periodic headache treatment could cause chronic headaches [1]. After the medication was discontinued, the headache symptoms improved, although some patients had withdrawal effects [2].

The so-called medication-overuse headache (MOH) or rebound headache is now recognized as the most common cause of secondary headaches and develops as a consequence of excessive and frequent use of analgesics for the acute treatment of primary headache disorders. MOH- can be diagnosed in patients with pre-existing primary headache, who have headaches occurring on 15 or more days per month and regularly overuse acute treatments for more than 10–15 days per month for three consecutive months [3].

The pathophysiological mechanisms that underpin MOH are not fully understood but likely represent the complex interplay between certain psycho-social factors [4,5] and the class of acute medication [6] that is overused in genetically predisposed individuals [7].

Although education remains the most important means of preventing development of MOH, the advent of drugs that target the calcitonin gene-related peptide (CGRP) system for both acute pain relief [8,9,10,11] and prevention of migraines [12,13,14,15,16,17] is likely to play an important role in the future management of this disabling headache disorder.

In this review article, we examine the following: the burden of MOH; who develops MOH; the pathophysiological mechanisms; and the treatment strategies.

## 2. Epidemiology

Studies indicate that, worldwide, up to 1–4% of the population overuses analgesics for the treatment of chronic pain conditions [18]. The prevalence of MOH in adults ranges between 0.5 and 2.6%, with higher rates of up to 7.6% reported in Russia [19]. Around 11 to 70% of people with chronic headaches, especially migraines, also have MOH [19]. Within our headache service at a large UK teaching hospital, a recent service evaluation indicated that approximately 30% of adult patients with a migraine at the time of referral also had an MOH.

An MOH is most common in adults aged 30–50 years old and affects women three to four times more than men [20]. MOH is less common in adolescents and people over 65 years old, with prevalence reported as 0.2–0.3% and 1.0–1.7%, respectively [19].

## 3. Risk Factors

MOH occur in people with a co-existent primary headache who overuse acute pain medications. Migraine is the most common primary headache disorder associated with the MOH and accounts for 78% of patients, while in 15%, there is a co-existent tension-type headache or, rarely, cluster headache [21] People taking analgesics for other chronic pain conditions such as rheumatoid arthritis or ulcerative colitis may develop an MOH if they have background of primary headaches, particularly migraines [22].

With the exception of Gepants and Ditans, all acute pain medications used to treat headaches have the potential to cause MOH. Triptans and opiates appear to carry the highest risk and can cause an MOH over a shorter time period. In one study, triptans were associated with increased headache frequency in those with more than 10 days of headache at baseline [23]. Mean time to the onset of an MOH was of a shorter duration in patients receiving triptans (1.7 years) or opiates (2.2 years) compared to other analgesics (4.8 years) [6]. Anti-inflammatory drugs such as non-steroidal anti-inflammatory drugs (NSAIDs) appear to be protective against the transition to chronic headaches in people with a low frequency of migraines (less than 10 days per month) but increase the risk in those with frequent headaches (over 10 days per month) [24]. Although different analgesics are associated with differing risks of developing MOH, the major predictor is a high headache frequency at baseline, regardless of medication history [24].

Headache patients with comorbid mental health conditions, including anxiety and depression, appear more susceptible to MOH. In one study, approximately 30% had a history of major depression disorder and 26% had a history of anxiety disorders [4]. Other associated psychiatric disorders which confer risk include the following: other mood disorders; obsessive-compulsive disorder; post-traumatic stress disorder; and drug dependence [4]. Others risk factors include the following: age below 50 years; low education level; chronic musculoskeletal or gastrointestinal complaints; smoking; physical inactivity; metabolic syndromes; high caffeine intake; and tranquillizer use [5]. Patients from ethnic backgrounds and with low levels of literacy would also appear to be at greater risk [25].

Certain genes probably increase the risk of developing an MOH. This is supported by the observation that the development of MOH is more likely if there is a family history of MOH or substance abuse [26]. Additionally, MOH appear to share similar mechanisms with drug addiction [27], and genetic variations are factors in susceptibility to drug addiction [28]. Polymorphic variants of the dopaminergic gene system or of other genes related to drug-dependence pathways appear to the most closely associated with MOH [7].

The overall risk of developing MOH is therefore complex and relates to the interplay between certain psycho-social factors and the class of acute medication that is overused in in genetically predisposed individuals [7].

## 4. Pathophysiology

The pathophysiology of MOH remains poorly understood but is likely to involve both the peripheral and central neuronal networks associated with the chronification of pain. Possible mechanisms of MOH development include the following: increased central sensitization of trigeminal nociceptive pathways; increased peripheral trigeminal sensitization; and increased cortical excitability.

*Increased central sensitization of trigeminal nociceptive pathways*: Animal experiments provide evidence of structural and functional alterations following chronic analgesic use including the upregulation of neuropeptides involved in the transmission of pain (CGRP, substance P, and nitric oxide synthase) in trigeminal ganglia; the expansion of receptive fields, and the decreased nociceptive threshold of central trigeminal neurons [29].

*Increased peripheral trigeminal sensitization*: Peripheral trigeminal sensitization of nociceptors in perivascular areas may play a role in MOH. Anti-CGRP antibodies have been found to be effective in decreasing the number of headache days per month and controlling pain in those with an MOH without phasing out of the acute causative analgesics. The targeted mechanism of anti-CGRP antibodies is the nociceptors in the peripheral vascular areas, as the antibodies cannot pass through the blood–brain barrier [30].

*Increased cortical excitability*: Cortical excitability is associated with the cortical spreading depression (CSD) that accompanies migraine aura and leads to the increased sensitivity of the trigeminal pain pathway. Animal studies showed that chronic exposure to abortive pain medications such as paracetamol or specific migraine medication affects cortical activity by depleting 5-hydroxytryptamine (5-HT) in the central system [31,32]. Animal imaging studies have demonstrated an early change in cortical and subcortical brain networks 7 days after sumatriptan administration, and the rat subjects also have CSD-like responses when triggered by bright light stress [33]. CSD events may represent a mechanism of MOH related to the chronic exposure of triptans and other analgesics.

## 5. Diagnosis

An MOH has no distinguishing features and there are no specific laboratory tests that support diagnosis. According to the International Classification of Headache Disorders (ICHD-3) [3], MOH can be diagnosed in patients with a pre-existing primary headache, who have a headache occurring on 15 or more days per month and regularly overuse acute or symptomatic treatments for more than 10–15 days per month for 3 consecutive months. For triptans and opioids, this is more than 10 days per month; for other simple analgesics, including paracetamol, aspirin and NSAIDs, this is more than 15 days per month.

The latest criteria [3] were modified from previous ICHD-2 criteria by removing the following two requirements: (1) headache resolves to its previous pattern within 2 months after the discontinuation of overused medication and (2) headache develops or markedly worsens during medication-overuse. The benefit of removing the first criterion is that clinicians can diagnose and initiate treatment for an MOH on the day of evaluation. However, there is ongoing debate around the removal of the second criterion due to concerns that it may lead to the overdiagnosis of an MOH.

In clinical practice, an MOH most often develops in those with a migraine (or less commonly in a tension-type headache) who report an increased frequency of headaches on a recent background of frequent acute medication use. MOH may be indistinguishable from the pre-existing headache. Diagnosis can be difficult because the transition from episodic to chronic migraines or tension-type headaches often induces a more regular use of analgesics. The diagnosis of an MOH can be made retrospectively, following drug withdrawal if the headache symptoms improve.

Although MOH are a common secondary headache disorder, it is important to consider the other secondary causes of chronic headaches before making a diagnosis, including life-threatening conditions such as mass-occupying or vascular lesions; intracranial hypertension; intracranial hypotension; systemic symptoms suggest malignancy or vasculitis; and obstructive sleep apnea. Patients need a careful physical examination to exclude hypertension; focal neurological deficits (including visual field deficits); and papilledema. Some may also need further investigation including neuroimaging if red flag signs are present [34]. Significant abnormalities on magnetic resonance imaging of the head are reported in 1.2% and 0.9% of migraine and tension-type headache patients, respectively [35].

## 6. Prevention

Preventing the development of an MOH through the education of patients and healthcare professionals remains the most important management strategy. Many patients with chronic headaches are not aware of the concept of MOH. In a recent study of patients with chronic migraines, 40% were unaware of the term “medication-overuse headache” [25]. This group was overrepresented by older patients and patients from ethnic minority groups, highlighting the need for greater education. Ideally, patients with frequent headaches should be warned about the potential risks associated with the increased use of acute medications before the development of an MOH.

## 7. Treatment Strategies

**Figure 1:**
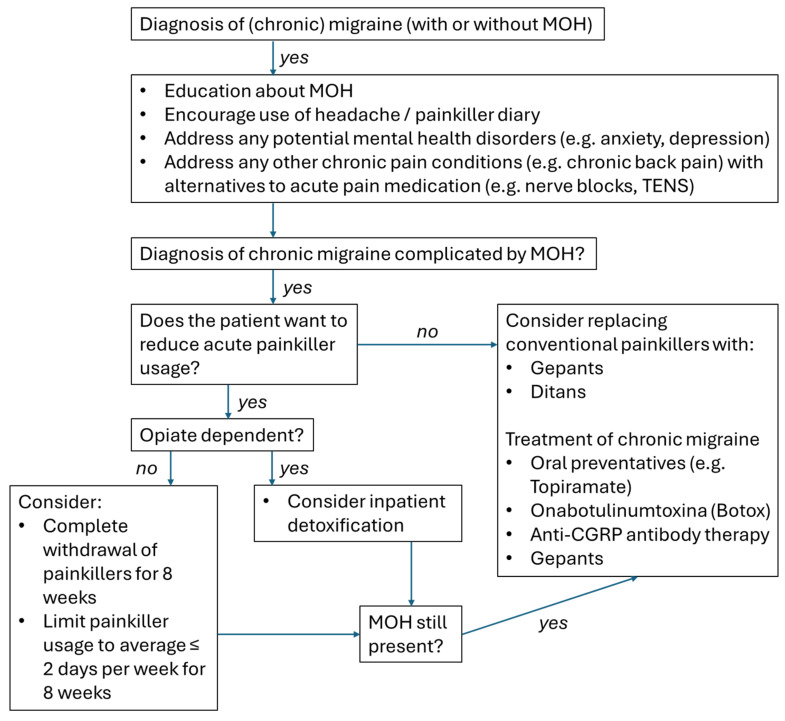
Management of chronic migraine complicated by medication-overuse headache. Key: MOH, medication-overuse headache; TENS, transcutaneous electrical nerve stimulation; CGRP, Calcitonin Gene-Related Peptide.

Once established, treating MOH can be difficult and, understandably, patients often display reluctance to reduce acute pain medication, which they have become reliant on in order to reduce headache burden and improve their level of function. In patients with an established MOH, there are two options: either reduction or complete withdrawal of acute pain medication, with or without a bridging treatment, or the addition of a migraine preventative in parallel; or accepting that acute pain medication reduction is not possible and starting a migraine preventative (Figure 1).

### 7.1. Advice to Patients

Before embarking on acute medication withdrawal, it is important to prepare patients for what lies ahead and to manage expectations. There is no agreed standard for what advice should include before acute medication withdrawal. This was highlighted in one study that showed no significant difference in headache outcomes between patient groups that had received structured or unstructured educational advice prior to withdrawal [36]. With that said, the following statements are likely to be useful in any discussions with patients: (1) The understanding that frequent use of acute medication to treat headache may be the contributing to more headaches; (2) the removal of acute medication is likely to improve headaches in the longer term; (3) headache improvement is unlikely to be complete; (4) the use of migraine preventative medication in parallel may reduce MOH; (5) the treatment of co-existing mental health condition (e.g., depression and anxiety) may offer additional benefits; (6) re-exposure to acute pain medication after successful withdrawal may cause the relapse of an MOH; (7) following the successful withdrawal of acute pain medication, the underlying headache needs to be treated; and 8) the successful treatment of MOH may take many weeks or months [37].

### 7.2. Education

Brief interventions to educate patients about MOH have been studied in different settings, including the general population, primary care, and dedicated headache centers [38,39,40] In each case, educational intervention has been shown to significantly reduce acute painkiller usage (38–72%) compared to baseline, resulting in a significant reduction in headache frequency.

### 7.3. Drug Withdrawal

Withdrawal of acute medication may be challenging in some patients, especially if there is a dependence on opioids and should therefore be individualized. Patients should be encouraged to reduce acute medication to below the levels known to cause an MOH. In the case of simple analgesics, this should be less than 15 days per month and, for triptans and opioids, less than 10 days per month.

Drug withdrawal requires patient buy-in and often puts a strain on doctor–patient relations. A realistic and pragmatic approach should therefore be adopted. Timing is crucial and drug withdrawal requires planning with the expectation that headaches will get worse before they get better. Patients should be encouraged to forewarn family members and employers of any potential disruption.

Abrupt withdrawal is advised in patients overusing simple analgesics or triptans and is invariably carried out in the out-patient setting. For patients who overuse opioids, drug withdrawal must be gradual and may require inpatient supervision. Such patients may develop a psychological or physiological dependence on opioids and an abrupt withdrawal could be dangerous.

During the withdrawal process, patients often see a transient increase in the frequency and severity of headaches, which typically lasts for 2–10 days. Associated withdrawal symptoms include nausea, vomiting, restlessness, sleep disturbance, and anxiety, all of which should be considered and managed separately where possible. One study showed that withdrawal symptoms were shorter in patients overusing triptans (mean of 4.1 ± 1.9 days) compared to simple analgesics (means 9.5 ± 3.5 days) [41].

In a Danish study, 175 patients with MOH had all their acute medication discontinued abruptly and were kept acute-medication-free for 8 weeks [42]. Up to 45% of patients reported a reduction in headaches by at least 50%, while 48% remained unchanged and 7% experienced an increase in headaches. The same group also compared the complete withdrawal of acute medication versus limiting the medication intake to a maximum of 2 days per week [43]. Complete withdrawal was more effective and resulted in a significantly greater reduction in headache days per month (−46% vs. −22%). Complete withdrawal of acute medication also resulted in the increased likelihood of reverting to an episodic headache pattern (70% vs. 42%) and a reduction in the number of migraine days per month (7.2 versus 3.6 days per month) after 6 months. In one study, inpatient detoxification was achieved in 48% more patients than those detoxified in out-patient clinics [44]. However, this approach is often limited by the access to hospital beds and the availability of a headache or pain specialist to oversee the withdrawal process.

The use of bridging or rescue therapies to reduce withdrawal symptoms has been studied. There are no high-quality data to support the use of corticosteroids or alternative painkillers (e.g., COX-2 inhibitors) in this process [45]. The use of supportive intravenous fluids [44,46], lidocaine infusions [47], and greater occipital nerve blocks [48] have all been shown to improve the chances of successful detoxification in some patients.

### 7.4. Migraine Prevention

Some clinicians may choose to start migraine prevention in parallel to acute medication withdrawal. In the COMOESTAS study, patients were allowed acute medication up to 2 days per week and started migraine prevention in parallel [49]. After 6 months, the monthly headache frequency was reduced by 58% compared to the baseline. A Danish trial concluded that patients receiving early migraine prevention during the withdrawal proceed resulted benefited more quickly [50].

Not all patients with migraines complicated by an MOH need to withdraw from acute headache medication to respond to migraine preventive medication. There is now a body of evidence from different trials supporting the use of various migraine preventatives, including topiramate [51,52], onabotulinumtoxinA [53], and anti-CGRP monoclonal antibodies therapies [54,55,56,57,58], in patients with chronic migraines complicated by an MOH.

## 8. Novel Treatments for Migraine

Gepants and Ditans are novel selective acute migraine medications that work by inhibiting the release of CGRP in trigeminovascular signaling pathways and, unlike convention acute painkillers for migraine, are unlikely to cause MOH. Gepants target the CGRP receptor [59], while Ditans target the 5-HT_1F_ receptor [60], which in turn leads to inhibition of CGRP release. For acute migraine treatment, Rimegepant, Ubrogepant, Vazegepant, and Lasmiditan have shown efficacy in reduced migraine pain with favorable tolerability and safety [8,9,10,11]. Additionally, Gepants play a role in migraine prevention. The use of daily Atogepant and alternate-day Rimegepant for twelve weeks significantly reduced the mean monthly migraine days, compared to the placebo [16,17]. Currently, there are no data indicating that the chronic regular use of Ditans or Gepants induces MOH, and therefore replacing the conventional painkillers for migraines with these novel drugs is likely to reduce the burden of MOH.

## 9. The Future: How to Prevent Development of an MOH

We believe that in many cases, the development of an MOH is potentially preventable through education, especially in those patients and healthcare professionals who are unaware of the condition and how it develops. In our experience, many patients, even when aware of MOH, do not record how many painkillers they use and, in cases of high headache frequency, well-established thresholds for the maximum recommended usage are often inadvertently crossed. More research is therefore required on how best to educate patients and healthcare professionals about MOH before it develops. With the advent of widescale smartphone usage, and the increased availability of mobile applications serving as electronic headache diaries, patients who are aware of MOH are likely to use painkillers less frequently.

## 10. Conclusions

MOH remain the most common cause of secondary headaches and is particularly problematic in patients with chronic migraines, many of whom also have mental health disorders. The pathogenesis of MOH is thought to relate to increased cortical excitability and the central and peripheral sensitization of the trigeminal nociceptive pathway. Prevention through the education of patients and healthcare professionals remains an important management strategy. Once established, MOH are significantly reduced by complete analgesic withdrawal or restriction to a maximum of 2 days per week. When this cannot be achieved the use of migraine prevention, especially OnabotulinumtoxinA and therapies that target the CGRP system, has been shown to be of benefit. The use of novel acute pain therapies for migraines, including Gepants and Ditans, which do not cause MOH, are likely to improve patient outcomes, although they are expensive compared to conventional acute treatments and therefore are not always readily accessible.
